# Travel to Asia is a strong predictor for carriage of cephalosporin resistant *E. coli* and *Klebsiella spp.* but does not explain everything; prevalence study at a Norwegian hospital 2014–2016

**DOI:** 10.1186/s13756-018-0429-7

**Published:** 2018-11-29

**Authors:** Laura Espenhain, Silje Bakken Jørgensen, Truls Michael Leegaard, Michaela Marie Lelek, Siri Haug Hänsgen, Britt Nakstad, Marianne Sunde, Martin Steinbakk

**Affiliations:** 1 Department of Antibiotic Resistance and Infection Prevention, Norwegian Institute of Public Health, PO Box 222 Skøyen, 0213 Oslo, NO Norway; 2 European Programme for Intervention Epidemiology Training (EPIET), European Centre for Disease Prevention and Control, (ECDC), Stockholm, Sweden; 3 Department of Clinical Microbiology and Infection Control, Akershus University Hospital, Lørenskog, Norway; 4 Institute for Clinical Medicine, Campus Ahus, University of Oslo, Nordbyhagen, Norway; 5 Department of Paediatrics and Adolescents Medicine, Akershus University Hospital, Lørenskog, Norway; 6 Department of Molecular Biology, Norwegian Institute of Public Health, Oslo, Norway; 7 Section for Food Safety and Emerging Health Threats, Norwegian Veterinary Institute, Nordbyhagen, Norway

**Keywords:** Prevalence, Beta-lactam resistance, Hospitals, university, Cross-sectional, Studies, Enterobacteriaceae, Epidemiology, Drug resistance, multiple

## Abstract

**Background:**

We aimed to estimate the prevalence of faecal carriage of extended-spectrum cephalosporin (ESC) resistant *E. coli* and *K. pneumoniae* (ESCr-EK) and vancomycin resistant enterococci (VRE) in patients upon hospital admission and identify factors associated with carriage to better target interventions and to guide empirical antibiotic treatment.

**Methods:**

Between October 2014 and December 2016, we recruited patients admitted to a Norwegian university hospital. A rectal swab and questionnaire covering possible risk factors for colonisation were collected upon admission. Isolates were characterized by phenotypic methods. ESCr-EK isolates were subject to whole genome sequencing. We calculated prevalence and adjusted prevalence ratios (aPR) using binomial regression.

**Results:**

Of 747 patients, 45 (6.0%) were colonised with ESCr-EK*,* none with VRE*.* The ESCr-EK isolates in 41 patients were multidrug resistant; no isolates were non-suceptible to meropenem. Prevalence of ESCr-EK was higher among travellers to Asia (aPR = 6.6; 95%CI 3.6–12; *p* < 0.001). No statistical significant difference in carriage was observed between departments, age or any other factors in the univariable analyses.

**Conclusions:**

The observed prevalence of ESCr-EK colonisation upon admission was in the same range but lower than that reported in similar studies from Europe. Travel to Asia was a strong predictor for colonisation of ESCr-EK to be considered when administering empirical antimicrobial treatment. As less than one third of colonised patients had travelled to Asia, and no other factors investigated were found to be strongly associated with carriage, these findings underscore that healthcare personnel must apply standard infection control precautions for all patients.

**Electronic supplementary material:**

The online version of this article (10.1186/s13756-018-0429-7) contains supplementary material, which is available to authorized users.

## Background

*Escherichia coli* and other *Enterobacteriaceae* frequently cause nosocomial and community acquired infections [1] and the burden due to infection with *Enterobacteriaceae* resistant to extended-spectrum cephalosporins (ESC) is increasing worldwide [2]. Infections are often preceded by colonisation of the gut with these bacteria. Among *Enterobacteriaceae*, resistance to ESC is often caused by production of extended-spectrum beta-lactamases (ESBLs) or plasmid mediated AmpC (pAmpC) [2]. In a hospital setting, carriage of *Enterobacteriaceae* resistant to ESC (ESCr-E) is not only a concern for the individual patient, but also for the surrounding patients as these bacteria can spread via the faecal-oral route between patients within the hospital [3]. In addition, plasmids conferring ESC resistance can be transferred between different bacterial strains and species. Outbreaks of ESBL-producing *E. coli* and *Klebsiella spp*. have been reported in healthcare institutions in Norway [4–6]. Yet another concern is that ESC-resistance often occurs along with co-resistance to important non-beta-lactam antibiotic classes [7], significantly limiting treatment options for these patients.

*Enterobacteriaceae* are part of the normal gut flora and pose a complex challenge as eradication is problematic [8]. If introduced within a hospital, infection prevention and control rely on strict compliance to standard precautions [3, 9]. The Norwegian Institute of Public Health (NIPH) recommends isolation of patients in general hospital wards if they carry carbapenemase producing *Enterobacteriaceae* or vancomycin resistant enterococci. Patients who carry ESC resistant *Enterobacteriaceae* should be isolated if admitted to departments with particularly vulnerable patients (neonatal, cancer or intensive care units) [10]. Even though the Nordic countries have a low prevalence of antimicrobial resistance among *Enterobacteriaceae*, resistance to ESC in *E. coli* and *Klebsiella ssp* in urine and blood specimens is increasing [11]. In order to better target interventions to prevent spread between patients and to guide empirical antibiotic treatment we aimed to estimate the prevalence of faecal carriage of ESC resistant *E. coli* and *K. pneumoniae* (ESCr-EK) and vancomycin resistant enterococci (VRE) in patients upon hospital admission, and to identify factors associated with carriage. Additionally we aimed to explore the diversity of ESCr-EK and determine the genetic background for resistance to ESC.

## Methods

### Design, setting and study population

The cross-sectional study was carried out at Akershus University Hospital, a secondary care facility located in a mixed urban and rural area east of the Norwegian capital Oslo. The catchment area covers around 10% (500,000 persons) of the Norwegian population. Trained nurses recruited patients upon admission. In the adult’s emergency department, recruitment took place on selected weekdays between October 2014 and July 2015, all patients presenting between 8 am and 11 am were invited to participate. In the children’s emergency department, all patients/parents admitted during selected weeks in the period between January 2015and July 2016 were invited to participate. From October 2015 to December 2016 recruitment was expanded to three surgical units (thoracic, urology, and orthopaedic) where all patients presenting for pre-surgical examination prior to elective surgery were invited to participate.

Patients with suspected acute coronary disease, in need of immediate intensive care, and adult patients unable to give informed consent were not recruited.

### Data collection

Consenting patients or parents of patients below 16 years of age completed a questionnaire with 26 questions, including demographics and possible risk factors for colonisation with ESCr-EK such as travel outside of the Nordic countries, antibiotic consumption, health care and medical devices within the 12 months prior to recruitment. Only information about risk factors prior to admission was collected. One rectal swab was collected at admission or maximum 24 h after admission.

### Microbiology

Rectal swabs were collected and transported in liquid medium (eSwab, Copan, Italy). The samples were stored at 4 °C for maximum 14 h, with subsequent plating on blood agar for growth control, and on two selective lactose agar plates with the addition of either 1 mg/ml cefotaxime or 1 mg/ml ceftazidime for detection of ESCr-EK, produced in-house. For the detection of vancomycin resistant Enterococci, we used Brilliance VRE plates (Oxoid, ThermoFisher Scientific, MA, USA). Morphologically distinct colonies from each selective plate were subcultured, and identified by MALDI-TOF mass spectrometry (MALDI-TOF, Bruker Daltonics, Bremen, Germany). Isolates of *E. coli* or *Klebsiella* spp. were frozen at -80 °C for later analysis. We did susceptibility disk testing and interpretation of clinical breakpoints according to EUCAST methodology (http://www.eucast.org/fileadmin/src/media/PDFs/EUCAST_files/Breakpoint_tables/Breakpoint_table_v_4.0.xls, 2017), and divided the antibacterial agents into six classes: Penicillins (Ampicillin), cephalosporins and penicillins in combination with betalactam-inhibitors (cefoxitin, cefotaxime, ceftazidime and piperacillin-tazobactam), carbapenems (meropenem), (fluoro)quinolones (ciprofloxacine and nalidixin), aminoglycosides (gentamicin), and folate-pathway inhibitors (trimethoprim-sulphamethoxazole). We defined multidrug resistance as non-susceptibility (i.e. resistant or intermediate susceptible) to three or more of the above antibiotic classes.

From each patient positive for ESCr-EK, one isolate was selected for whole genome sequencing (WGS). If the patient carried both ESC resistant *E. coli* and *Klebsiella spp*., one isolate per species was included for WGS. Furthermore, if a patient carried several morphologically different strains of the same species, and if the strains had different antimicrobial susceptibility patterns (indicative of polyclonal carriage), one representative isolate for each pattern was included for WGS. WGS was done with Illumina technology (Illumina, San-Diego, USA), and data were analysed on the publicly available platform at the Center for Genomic Epidemiology [12]. Default threshold settings were used for MLST and ResFinder [13, 14].

### Statistical analysis

We expressed the prevalence of ESCr-EK upon admission as the number of patients positive for ESCr-EK per 100 patients enrolled in the study. For the descriptive analysis, and to evaluate independent factors associated with colonisation we performed uni- and multivariable binominal regression analyses. We grouped countries into five regions (Europe, Asia, Americas, Africa and Australia). The category “Asia” included countries in the Middle-East as well as Central and East Asia (listed in Additional file 1). In the multivariable analyses variables with a *p*-value of < 0.2 were included in the model one by one starting with the factor with the highest prevalence ratio. Model fit was evaluated using the likelihood-ratio test. *P*-values < 0.05 were considered significant. Health care outside of the Nordic countries, included in the national screening guidelines, was included in the final model irrespective of level of significance.

### Human subject protection

Informed consent was obtained from each patient or parents of patients below 16 years of age. The Regional Committees for Medical and Health Research Ethics (REC 2012/2234) and the hospital’s privacy protection officer approved the study protocol. The study was funded by the NIPH.

## Results

### Participation

In total, 747 rectal swabs accompanied by a completed questionnaire were collected from patients upon admission. Among the participants, 391 (52%) participants were male, ranging from 43% in the orthopaedic unit to 62% in the urology unit. Twenty per cent were below three years of age and 39% were 65 years or older. An estimate of 84% of patients invited to participate accepted.

### Microbiology

#### Patients

Of the 747 rectal swabs, 45 (6.0%) contained one or more isolate resistant to cefotaxime and/or ceftazidime. *E. coli* was found in 43 of the 45 samples and *K. pneumoniae* in five, i.e. both ESC resistant *E. coli* and *K. pneumoniae* were identified in three samples. Co-resistance to fluoroquinolones, piperacillin-tazobactam and gentamicin was observed in ESCr-EK isolates from 39, 11 and 10 patients, respectively. The ESCr-EK isolates in 41 patients were multidrug resistant. No patients carried isolates non-susceptible to meropenem. Carriage of vancomycin resistant enterococci was not detected in any of the participants.

#### Isolates

In total, 57 ESCr-EK isolates from the 45 ESCr-EK carriers were subject to WGS. Among these, 51 isolates from 42 patients harboured one or more ESBL genes of the *bla*_CTX-M_ groups (5.6% of all patients) and five isolates from four patients harboured pAmpC genes (*bla*_DHA-1_ or *bla*_CMY-42_) (Table 1). In one patient both *bla*_CTX-M_ and *bla*_DHA-1_ were identified in different isolates. One isolate harboured both *bla*_CTX-M-15_ and *bla*_CMY-42_. In two isolates, one *E. coli* and one *K. pneumoniae*, ResFinder v3.0 (accessed July 27th 2018) did not detect any resistance mechanism to explain ESC resistance.

**Table 1:** Genotypes of ESCr-EK isolates (*n* = 52 E.coli and 5 *K. pneumoniae*) from 45 ESCr-EK carriers

Enzyme	gene identified	*E. coli*	*K. pneumoniae*
ESBL	*bla*_CTX-M-15_	28	1
	*bla*_CTX-M-55_	6	
	*bla*_CTX-M-27_	6	
	*bla*_OXA-1_^a^	8	
	*bla*_CTX-M-14(+b)_	5	
	*bla*_CTX-M-1_	4	
	*bla*_CTX-M-3_	1	1
	*bla*_TEM-33_	1	
pAmpC	*bla*_DHA-1_	2	2
	*bla*_CMY-42_	1	
Uncertain	None detected	1	1

^a^*bla*_-OXA-1_ always occurring in isolates also harbouring *bla*_CTX-M-15_

Among the 57 isolates that were subject to WGS, 25 different MLSTs were identified in *E.coli* and two in *K. pneumoniae*. One fourth of the *E. coli* [14] belonged to ST131 (Table 2). Three patients carried several *E. coli* strains with different MLSTs. Nine MLSTs (34, 83, 93, 99, 349, 398, 410, 485, and 617) were found only in patients who had solely travelled to Asia.

**Table 2:** Distribution of MLST types ESCr-EK isolates (*n* = 52 E.coli and 5 K. pneumoniae) from 45 ESCr-EK carriers

MLST type	*E. coli*	*K. pneumoniae*
131	14	
38	5	
405	4	
10	2	
34	2	
69	2	
83	2	
357	2	
648	2	
Unknown MLST^a^	2	3
Other MLST types^b^	15	2
Total	52	5

^a^MLST not resolved

b*E. coli*: MLST48, 73, 93, 99, 167, 394, 398, 410, 485, 617, 1163, 1193, 1611, 1722, 3036. *K. pneumoniae:* MLST37, 1427

#### Epidemiology

In the univariable analyses (Table 3), we observed a significantly higher prevalence of ESCr-EK in patients who had travelled to Asia (33%) compared with those who had not travelled to Asia (4.5%), in patients who had travelled outside of the Nordic countries (11%) compared to patients who had not (3.9%), and in patients who had received health care outside the Nordic countries (14%) compared to those who had not (5.6%). A higher, but non-significant, prevalence was observed in patients born outside Norway (11%) compared to patients born in Norway (5.9%), and in those who had travelled to Africa (18%) compared to those who had not (5.8%). No statistical significant difference in carriage was observed between departments of recruitment or age in years or age group or any other factors in the univariable analyses (Table 3).

**Table 3:** Number of patients, carriers, percent carriers, crude prevalence ratio (PR) and *p*-values from the univariable binominal regressions by risk factors for ESCr-EK carriage up to 12 months prior to admission (*n* = 747)

			Carriage of ESCr-EK			
		Total no. of patients	No. of patients	(%)	Prevalence ratio (PR)	*p*-value
		747	45	(6.0%)		
Demography	Female	356	16	(4.5%)		
	Male	391	29	(7.4%)	1.65	0.10
	0-17 year	222	11	(4.9%)		
	18 - 64 years	233	18	(7.7%)	1.81	0.10
	65 + years	292	16	(5.5%)	1.17	0.67
	Born in Norway	675	40	(5.9%)		
	Not born in Norway	45	5	(11.1%)	1.88	0.16
	Parents born in Norway	593	35	(5.9%)		
	One or both parents not born in Norway	114	8	(7.0%)	1.19	0.65
Department of inclusion	Emergency department (adult)	122	11	(9.0%)		
	Emergency department (children)	222	11	(4.9%)	0.56	0.14
	Thoracic unit	194	11	(5.7%)	0.63	0.26
	Orthopaedic unit	141	7	(5.0%)	0.56	0.20
	Urology unit	68	5	(7.3%)	0.81	0.69
Exposures 12 months prior to admission	No travel outside of the Nordic countries^a^	512	20	(3.9%)	0.37	0.00
	Travel to Europe (vs. no travel to Europe)	175	11	(6.3%)	1.1	0.87
	Travel to America (vs. no travel to America)	22	1	(4.6%)	0.75	0.77
	Travel to Africa (vs. no travel to Africa)	11	2	(18.2%)	3.11	0.08
	Travel to Asia (vs. no travel to Asia)	51	15	(29.4%)	6.82	0.00
	No health care (vs. health care)	499	32	(6.4%)	0.82	0. 55
	Admitted to a Norwegian hospital^b^	224	11	(4.9%)	0.81	0.55
	Medical examination or treatment outside the Nordic countries^c^	28	4	(14.3%)	2.54	0.05
	Work in health care (vs. other type of job)	38	3	(7.9%)	0.95	0.94
	Work in farming	11	0	(0.0%)	-	-
	Other type of job	193	16	(8.3%)		
	No contact to animals at work	188	17	(9.0%)		
	Contact to animals at work	22	1	(4.5%)	0.50	0.49
	No permanent urinary catheter	668	40	(6.0%)		
	Permanent urinary catheter	10	0	(0.0%)	-	-
	No medical devices put through skin/mucosa before admission	639	39	(6.1%)		
	Medical devices put through skin/mucosa	46	2	(4.3%)	0.71	0.63
	No wound or skin infections	616	34	(5.5%)		
	Wound or skin infections	76	6	(7.9%)	1.43	0.40
	No antibiotic consumption	515	28	(5.4%)		
	Antibiotic consumption	232	17	(7.3%)	1.35	0.31

^a^(vs. travel outside of the Nordic countries)

^b^(vs. not admitted to a Norwegian hospital)

^c^(vs. no medical examination or treatment outside the Nordic countries)

In the multivariable analyses, travel to Asia was the only factor associated with carriage. Health care outside the Nordic countries was also included in the reported model as it is part of the national screening recommendations. The binominal regression analysis showed that travellers to Asia had a significantly higher prevalence of ESCr-EK carriage compared to those who had not travelled (PR 6.1; 95% CI 3.4–11; *p* < 0.001) (Table 4). Travel to Asia was primarily to Thailand (*n* = 17), Turkey (*n* = 17), and Pakistan (*n* = 6).

**Table 4:** Number of patients, carriers and adjusted prevalence ratio (aPR), 95% CI and *p*-values from the multivariable binominal regression model by risk factors for ESCr-EK carriage up to 12 months prior to admission (*n* = 741)

	Total	Number of ESCr-EK carriers	aPR	95% CI	*p*-value
Number of patients	741	44			
Travel to Asia the past 12 months	50	14	6.1	(3.43–11)	< 0.001
Medical examination or treatment outside the Nordic countries	28	4	1.7	(0.74–3.9)	0.212

*aPR* Adjusted prevalence ratio

## Discussion

In this study, including 747 patients from two emergency departments and three surgical units, we found no *E.coli* or *Klebsiella spp.* resistant to carbapenems, and no vancomycin resistant enterococci. The prevalence of colonisation of ESCr-EK upon admission was 6.0%. A major predictor for colonisation was travel to Asia. However, the majority of colonised patients had not been to Asia 12 months prior to admission.

The observed prevalence of ESCr-EK (6.0%) and ESBL-EK colonisation (5.6%) at admission was in the same range but lower than reported in similar studies from Germany (7.5–9.5%) [15], the Netherlands (8.2%) [16], and Israel (10.8%) [17] and slightly higher than previously reported in healthy individuals in Sweden (4.7%) [18] and Norway (4.9%) [19]. Varying methods and definitions challenge direct comparison between studies and this may, at least in part, explain these differences between countries. Other explanations may be differences in age, ethnicity, co-morbidity, previous hospital admissions or a true lower prevalence upon admission in the Norwegian population compared to the prevalence in the Netherlands, Germany or Israel. To our knowledge, there has been no previous Norwegian studies of ESBL prevalence in patients at admittance to hospital.

Self-reported use of antibiotics has been found to be a risk factor for ESC-E colonisation at admission in Germany [15]. We could not confirm this finding, nor could studies from the Netherlands [16] and Sweden [18]. Differing antibiotic-prescription policies may be one explanation for this. It was not possible to perform stratified analyses on antibiotics class as one third of participants who had used antibiotics 12 months prior to the admission did not indicate which class.

Consistent with other studies, visiting Asia within 12 months prior to faecal sampling, was associated with ESCr-EK colonisation (PR 6.8, *p* < 0.001). Prevalence ranging from 13 to 45% has been found in Swedish [18, 20] and Danish [21] travellers to Asia. In accordance with our study, none of these studies found carbapenem-resistant *Enterobacteriaceae.* However, the methods we used were not optimised to detect carbapenemases like OXA-48, which do not also confer resistance to cefotaxime or ceftazidime. The higher prevalence in patients who had travelled to Asia is also consistent with results from prevalence studies from Asia (Pakistan [22], Thailand [23–25], Turkey [26]) on different populations and types of specimens, where a prevalence of up to 40–77% has been reported even in healthy adults and food products.

This study covers a convenience sample of a population utilizing the hospital. We recruited patients from two emergency departments and three elective surgery units, and a wide range of ages from infancy to elderly patients. It was not practically feasible to recruit critically ill patients admitted to intensive care units in the hospital. Hence, we do not describe the prevalence of ESCr-EK in units with patients at the higher risk for a severe outcome if colonised with resistant bacteria. The prevalence tended to be higher amongst patients who reported having received health care outside of the Nordic countries. However, there were not enough patients in this category to give sufficient power to quantify a difference. The data collected was not detailed to a level allowing for stratification on type of health care (hospital admission, dental care, primary health care, etc.).

The genetic mechanisms behind ESC resistance was mainly ESBL production due to *bla*_CTX-M_. Only two ESCr-EK could not be explained by ESBL or pAmpC production. We found a large diversity of MLSTs in the 45 patients carrying ESCr-EK, making comparison with previous findings challenging. Sixteen different MLSTs were found in patients who had travelled to Asia, nine of these were found in travellers who had only been in Asia. However, many of these MLSTs represent well-known clones that have been found in various sources in several countries, including in Norway [7]. The dominant ESBL allele was *bla*_CTX-M-15_ which has emerged and disseminated worldwide [27] and has previously been found in travellers returning from all over the world [20], and in environmental samples from our area [7]. The ESBL allele *bla*_CTX-M-55_ has been found to be one of the most commonly encountered ESBL-encoding genes in *Enterobacteriaceae* from food animals in China and has also been found in food animals in Europe together with other CTX-M types [28, 29] suggesting the potential importance of animal-to-human spread via contaminated food [20].

In our study, travel to Asia was primarily to Thailand and Pakistan. We expect that travel to other high endemic areas in Asia like India, or to Africa as found in other studies [18, 20], might also be a risk factor for colonisation as also indicated in our study. All the ESCr-EK isolates from patients who had travelled to Asia were multidrug resistant.

Urinary tract infections and bacteraemia are often preceded by colonisation of the gut [17, 30]. As more than one third of patients who had travelled to Asia within the last 12 months carried ESCr-EK, we would recommend that these patients receive empiric antimicrobial treatment effective against ESCr-EK if they present with signs of serious infections of possible Gram-negative origin. According to findings in several studies, and repeated here, ESCr-EK are often multidrug resistant, and carbapenems is probably the safest choice for empirical treatment [31]. Treatment should always be adjusted and, if possible, de-escalated as soon as culture results are available [32, 33].

Certain ESCr-EK phylogroups or subclones have been linked to increased potential to cause severe infections because of higher virulence [18]. More knowledge of the more virulent phylogroups or subclones and risk factors for colonisation would allow targeted screening/treatment guidelines. As of today, the national guidelines for ESCr-EK infection control and prevention do not differentiate between MLSTs.

## Conclusion

The prevalence of colonisation of ESCr-EK upon admission was 6.0%, none of the isolates identified from the patients produced carbapenemases and we did not identify any VRE, supporting other surveillance studies which indicate that carbapenemase-producing *E. coli* and *Klebsiella and* VRE are still very uncommon in the Norwegian population [11, 19]. Travel to Asia was a strong predictor for colonisation of ESCr-EK to be considered when administering empiric antimicrobial treatment. Less than one third of the colonised patients had travelled to Asia, and no other factors investigated were found to be strongly associated with carriage. For infection prevention and control, these findings underscore that health care personnel cannot rely solely on risk assessment and screening results but must apply standard infection control precautions for *all* patients.

## Additional file

Additional file 1: Countries of Asia and number of participants who have travelled there (*n* = 747). (DOCX 29 kb)
