# Supplementary material for: Travel to Asia is a strong predictor for carriage of cephalosporin resistant E. coli and Klebsiella spp. but does not explain everything; prevalence study at a Norwegian hospital 2014–2016
Source: Antimicrob Resist Infect Control. 2018 Nov 29;7:146. doi: 10.1186/s13756-018-0429-7 (PMC6262960; doi:10.1186/s13756-018-0429-7)
Supplement: Supplementary file 1 — Countries of Asia and number of participants who have travelled there (n = 747). (DOCX 29 kb) [file 13756_2018_429_MOESM1_ESM.docx]

*Additional material 1 : Countries of Asia and number of participants who have travelled there (n=747)*

| **Country** | **Number of patients*** |
| --- | --- |
| Thailand | 17 |
| Turkey | 17 |
| Pakistan | 6 |
| Iran | 2 |
| Israel | 2 |
| Jordan | 2 |
| Sri Lanka | 2 |
| Vietnam | 2 |
| Myanmar | 1 |
| China | 1 |
| Hong Kong | 1 |
| Japan | 1 |
| Libanon | 1 |
| Malaysia | 1 |
| Saudi-Arabia | 1 |
| Afganistan | 0 |
| Armenia | 0 |
| Aserbajan | 0 |
| Bahrain | 0 |
| Bangladesh | 0 |
| Bhutan | 0 |
| Brunei | 0 |
| Cambodia | 0 |
| Philippines | 0 |
| United Arab Emirates | 0 |
| Georgia | 0 |
| India | 0 |
| Indonesia | 0 |
| Iraq | 0 |
| Kazakhstan | 0 |
| Kyrgyzstan | 0 |
| Kuwait | 0 |
| Laos | 0 |
| Maldives | 0 |
| Mongolia | 0 |
| Nepal | 0 |
| North Korea | 0 |
| Oman | 0 |
| Qatar | 0 |
| Singapore | 0 |
| Sri Lanka | 0 |
| South Korea | 0 |
| Syria | 0 |
| Tajikistan | 0 |
| Taiwan | 0 |
| Turkmenistan | 0 |
| Uzbekistan | 0 |
| Yemen | 0 |

*Some patients have visited several countries
